# Buckling Analysis on Resin Base Laminated Plate Reinforced with Uniform and Functional Gradient Distribution of Carbon Fiber in Thermal Environment

**DOI:** 10.3390/polym15092086

**Published:** 2023-04-27

**Authors:** Xiaoqiang Zhou, Qingquan You, Yuan Gao, Fenfei Hua, Wanbiao Fu, Qingyang Huang, Yuanfang Wang

**Affiliations:** 1 Department of Mechanics, School of Aerospace Engineering, Huazhong University of Science and Technology, Wuhan 430074, China; qq_you@hust.edu.cn (Q.Y.);; 2 Hubei Key Laboratory of Engineering Structural Analysis and Safety Assessment, Wuhan 430074, China; 3 Aerospace Research Institute of Materials & Processing Technology, Beijing 100076, China; 4 Railway Locomotive and Vehicle Institute, Wuhan Railway Vocational College of Technology, Wuhan 430205, China

**Keywords:** carbon fiber reinforced composite, functionally graded materials, classical laminate plate theory, buckling, thermal effect

## Abstract

The present paper aims to investigate the buckling load of functionally graded carbon-fiber-reinforced polymer (FG-CFRP) composite laminated plates under in-plane loads in a thermal environment. The effective material properties of the CFRP composite are calculated by the Mori–Tanaka homogenization method. The theoretical formulations are based on classical laminate plate theory (CLPT) and the von Kármán equations for large deflections. The governing equations are derived based on the principle of virtual work and then solved through the Navier solution. Results are obtained for the critical buckling load and temperature effect of a simply supported plate subjected to in-plane loading. A detailed numerical study is conducted to provide important insights into the effects of the functionally graded carbon fiber (CF) distribution pattern and volume fraction, total number of layers, temperature, geometrical dimension and lamination angle on the buckling load of functionally carbon-fiber-reinforced composite plates. Finally, the validation is compared with the Reddy and finite element analyses, which show consistency with each other.

## 1. Introduction

Composite material refers to the combination of two or more kinds of materials with different properties or different structures, usually composed of matrix materials and reinforcing agents, and composites with carbon fibers as the reinforcement are called carbon-fiber-reinforced polymer (CFRP) composites. Carbon-fiber-reinforced polymers are composite materials that rely on carbon fiber for strength and stiffness while polymer provides a cohesive matrix to protect, hold the fibers together and provide some toughness. In recent years, carbon-fiber-reinforced, composite, laminated plate structures have been widely used in the aerospace [1], marine, automobile, architectural [2] and other engineering industries due to their superior characteristics, such as high strength and stiffness, low weight and high fatigue resistance [3,4,5]. For example, applications in aerospace may involve aircraft wing boxes, horizontal and vertical stabilizers, and wing panels [6]. When such structures are subjected to various types of loadings, buckling happens locally, such as material failure modes, and/or globally. The investigation of the behavior of structures under mechanical and thermal loading is a challenging task.

Approximation models for buckling problems usually employ numerical, analytical or semi-analytical approaches to evaluate the critical buckling load of structures, in which the Rayleigh–Ritz method [7], Galerkin method [8,9], finite strip method [10,11,12,13], etc. are commonly used. In most cases, the Ritz method is able to derive explicit results for the buckling load. Vescovini et al. [14] used the Ritz method for a free vibration and buckling analysis of composite plates; the Ritz approximation was applied to models based on both the classical lamination theory and a more advanced variable-kinematic formulation. Feng et al. [15] used the theorem of minimum potential energy and the Ritz method based on orthotropic plate theory to investigate the elastic buckling behaviors of trapezoidal corrugated-steel shear walls. Eirik et al. [7] developed an analytical model for a buckling analysis of stiffened panels, and the efficiency of the calculations was high. The Galerkin method is another effective algorithm for solving differential equations, and it can also be used to establish an eigenvalue problem for linear buckling analysis. Fiorenzo and Erasmo [8] investigated the accuracy of plate theories for buckling and vibration analysis, and the results were obtained based on both the Ritz and Galerkin methods. When the boundary terms were zero, then the two methods led to the same results. Jaberzadeh et al. [9] presented the solution for elastic and inelastic local buckling using the Galerkin method. Wang et al. [16] proposed the multiterm Kantorovich–Galerkin method to investigate the buckling and free vibration behavior of thin composite plates with the classical plate theory. In an analysis of shell structures, the finite strip method (FSM) combines the merits of both analytical and numerical methods and can be considered an efficient method to predict buckling loads. Dawe and Yuan [17] gave a description of the B-spline finite strip method for predicting the buckling stresses of rectangular sandwich plates, which allowed the efficient prediction of buckling stresses for both overall modes and highly localized, wrinkling-type modes. Ovesy and Assaee [10,11,12,13] developed a nonlinear, multiterm, finite strip method for the post-buckling analysis of thin-walled, symmetric, cross-ply laminated plates under uniform end-shortening. Their method was based on solving von Kármán’s compatibility equation to obtain mid-plane stresses and displacements, and then, by invoking the principle of the minimum potential energy, deriving equilibrium equations for finite strips. Pandit et al. [18] used an improved higher-order zigzag theory to study the buckling of laminated sandwich plates. Falkowicz [19] investigated the effect of the localization and geometric parameters of cut-outs on the buckling load using the finite element method. Debski et al. [20] investigated the effect of an eccentric compressive load on the stability, critical states and load-carrying capacity of thin-walled composite Z-profiles. Wysmulski [21] investigated the postbuckling behavior of eccentrically compressed, composite channel-section columns. Thus, more complex structures can be analyzed with numerical, analytical or semi-analytical approaches.

Functionally gradient composites, in which the material properties are graded but continuous, especially along the thickness direction, are heterogeneous composites in nature. The definition is the gradation in its material properties by changing the volume fraction of its constituent materials [22,23,24]. The incorporation of two different materials using gradients enhances the mechanical properties to withstand high temperatures, as the graded thermal barrier eliminates the stress concentration issue. These unique properties have been widely used in the aerospace, civil, mechanical and biomedical engineering fields [25,26,27]. Hu et al. [28] presented new analytic solutions for the buckling of non-Lévy-type, carbon nanotube (CNT)-reinforced, composite rectangular plates and the buckling problems of cantilever, free, and clamped plates. Lei et al. [29,30] used the element-free kp-Ritz method to conduct a buckling analysis of functionally graded composite laminated plates under various in-plane mechanical loads. A meshless model of variable-stiffness composite (VSC) plates was developed using a radial basis point interpolation method based on the naturally stabilized nodal integration scheme, and the buckling behavior of the VSC plates with elliptical cutouts was investigated [31]. The authors concluded that changes of the CNT volume fraction, plate width-to-thickness ratio, plate aspect ratio, temperature, boundary conditions and loading conditions have pronounced effects on the buckling strength of various types of carbon-nanotube-reinforced composite (CNTRC) plates. Moreover, it is worth noting that the type of distribution of CNT also significantly affects the buckling strength of CNTRC plates.

Shen et al. [32,33,34] showed that the material properties of the composite are affected by the variation of the temperature. As a result, a careful evaluation of the effects of thermal expansion is required to find the property and extent of their deleterious effects upon performance. Whitney and Ashton [35] first developed laminated plate equations, which include the effect of thermal strains. Shen et al. [34] proposed a perturbation technique to determine buckling loads and postbuckling equilibrium paths—and the governing equations of a laminated plate are based on Reddy’s higher-order shear deformation plate theory, which includes hygrothermal effects—and then presented an investigation on the nonlinear bending of functionally graded, graphene-reinforced, composite (FG-GRC) laminated plates resting on an elastic foundation and in a thermal environment [30]. The results of Shen and Zhang [36,37] show that the FG-X gradient arrangement can significantly improve the compressive buckling load of plates. Song et al. [38] presented compressive buckling analyses of functionally graded, multilayer, graphene nanoplatelet/polymer composite plates. Thai et al. [39] reported a NURBS formulation for free vibration, buckling and static bending analyses of multilayer, functionally graded, graphene-platelets-reinforced composite plates. Wu et al. [40] used fast converging finite double Chebyshev polynomials to investigate the post-buckling response of the functionally graded materials plate. Zaitoun et al. [41] presented the buckling response of FG “sandwich plate” on a viscoelastic foundation and exposed it to hygrothermal conditions. They confirmed that the characteristics of buckling were significantly affected by the temperature increase, character of the in-plane boundary conditions, transverse shear deformation, aspect ratio of the plate, total number of plies, fiber orientation, fiber volume fraction and initial geometric imperfections.

The present paper aims to investigate the buckling load of functionally graded CFRP plates under in-plane loads in a thermal environment. The effective material properties of the CFRP composite are calculated by the Mori–Tanaka homogenization method. The theoretical formulations are based on the classical laminate plate theory. The governing equations are derived based on the principle of virtual work and then solved through the Navier solution. Results are obtained for the critical buckling load and temperature effects of a simply supported plate subjected to in-plane loading. Detailed numerical studies are conducted to provide important insight into the effect of the CF distribution pattern and volume fraction, total number of layers, temperature, geometrical dimension and lamination scheme on the buckling load of FG-CFRP composite plates. This paper innovatively arranges the volume fraction of carbon fibers to be functionally graded distributed along the thickness direction.

## 2. Problem Formulation

**Figure 1:**
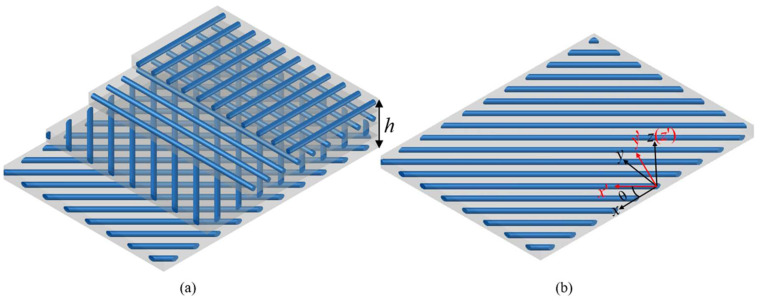
Structure of CFRP layers: (**a**) ply stacking sequence of the CFRP layers; (**b**) coordinate system transformation.

Part of the structure of CFRP composite plate is presented in Figure 1. The carbon fiber is laid with a specific direction in each layer. Its length and width are *a* and *b*, respectively. A cartesian coordinate system (*x*, *y*, *z*) is set on the mid-plane of the plate, defined by *z* = 0, where *z_n_* and *z_n_*_−1_ are the top and the bottom z-coordinates of the *n*th layer. The thickness of the plate is *h*.

**Figure 2:**
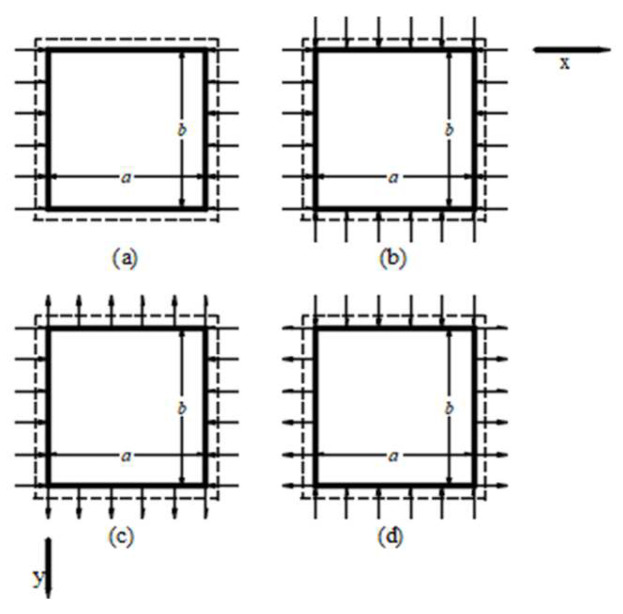
(**a**) Uniaxial compression, (**b**) Biaxial compression, (**c**) *k*_0_ < 0, *N*_cr_ > 0, (**d**) *k*_0_ = −1, *N*_cr_ < 0.

The geometry of the plate and loading condition are shown in Figure 2: $k_{0}=N_{y}^{0}/N_{x}^{0}$. Both positive and negative values of *k*_0_ are shown in Figure 2. The dotted line indicates simple support.

Various micromechanics models have been developed to determine the effective properties of fiber-reinforced composites. Herein, the Mori–Tanaka homogenization approach [39], which uses the average behavior of the matrix and fiber materials, is adopted. Considering transversely isotropic carbon fibers embedded in the isotropic matrix, the resulting properties of the CFRP composite plate can be expressed as in [42].

(1) $$E_{11}=V_{F}E_{11}^{F}+\left(1-V_{F}\right)E^{M}+2\xi_{1}V_{F}\left(1-V_{F}\right){\left(v_{12}^{F}-v^{M}\right)}^{2}$$

(2) $$E_{22}=\frac{E_{11}/\left(1-{\left(v^{M}\right)}^{2}\right)}{\frac{1}{1-{\left(v^{M}\right)}^{2}}+2V_{F}\frac{E_{11}}{\xi_{2}}\left[1+v_{23}^{F}-\frac{E_{22}^{F}}{E^{M}}\left(1+v^{M}\right)\right]+\xi_{1}V_{F}\frac{E_{11}^{F}}{E^{M}}\left(\frac{1+v^{M}}{E^{M}}-\frac{2}{E_{11}^{F}}+\frac{1-v_{23}^{F}}{E_{22}^{F}}\right)}$$

(3) $$v_{12}=v^{M}+\frac{2\xi_{1}V_{F}}{E^{M}}\left(v_{12}^{F}-v^{M}\right)\left[1-{\left(v^{M}\right)}^{2}\right]$$

(4) $$G_{12}=\frac{E^{M}}{2\left(1-V_{F}\right)\left(1+v^{M}\right)}\left\{1+V_{F}-4V_{F}{\left[1+V_{F}+2\left(1-V_{F}\right)\frac{G_{12}^{F}}{E^{M}}\left(1+v^{M}\right)\right]}^{-1}\right\}$$

(5) $$G_{23}=\frac{E^{M}}{2\left(1+v^{M}\right)+V_{F}{\left[\frac{1-V_{F}}{8\left[1-{\left(v^{M}\right)}^{2}\right]}+\frac{1}{E^{M}/G_{23}^{F}-2\left(1+v^{M}\right)}\right]}^{-1}}$$

where

(6) $$\xi_{1}={\left\{-\frac{2\left(1-V_{F}\right){\left(v_{12}^{F}\right)}^{2}}{E_{11}^{F}}+\frac{\left(1-V_{F}\right)\left(1-v_{23}^{F}\right)}{E_{22}^{F}}+\frac{\left(1+v^{M}\right)\left[1+V_{F}\left(1-2v^{M}\right)\right]}{E^{M}}\right\}}^{-1}$$

(7) $$\xi_{2}=E_{22}^{F}\left(3+V_{F}-4v^{M}\right)\left(1+v^{M}\right)+\left(1-V_{F}\right)E^{M}\left(1+v_{23}^{F}\right)$$

in which *V*_F_ is the volume fraction of fiber; E, G and *v* denote the Young’s modulus, shear modulus and Poisson’s ratio, respectively; and the superscript F and M signify the fiber and matrix. Consequently, the effective material properties of CFRP are functions of the CF volume fraction.

**Figure 3:**
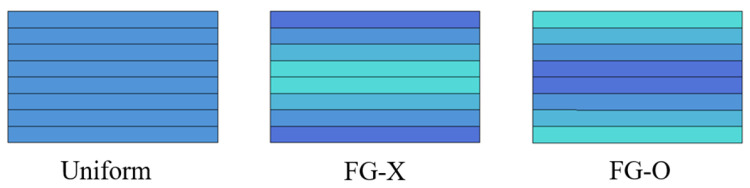
Functionally gradient carbon-fiber-reinforced polymer plates.

A functionally graded CFRP composite plate with the thickness *h* and length *a* is considered. The plate includes *N*_L_ layers, with the same thickness of each layer *h/N*_L_. CFs are assumed to be solid fillers uniformly dispersed in the polymer matrix of each layer, with a change of the CF volume fraction from layer to layer through the thickness of the plate. Three CFs’ distribution patterns, including uniform, FG-O and FG-X, are considered and plotted in Figure 3.

The volume fraction *V*_F_ follows a simple law:

(8) $$V_{F}^{\left(k\right)}=\left\{ \begin{array}{ll} V_{F}^{*} & \mathrm{Uniform}\\ 2V_{F}^{*}\frac{\left|2k-1-N\right|}{N} & \;\mathrm{FG}-X\\ 2V_{F}^{*}\left(1-\frac{\left|2k-1-N\right|}{N}\right) & \;\mathrm{FG}-O \end{array}\right.$$

where *N* is the number of layers, $V_{F}^{*}$ is the average of the volume fraction; $V_{F}^{\left(k\right)}$ is the volume fraction of the *k*th layer.

**Figure 4:**
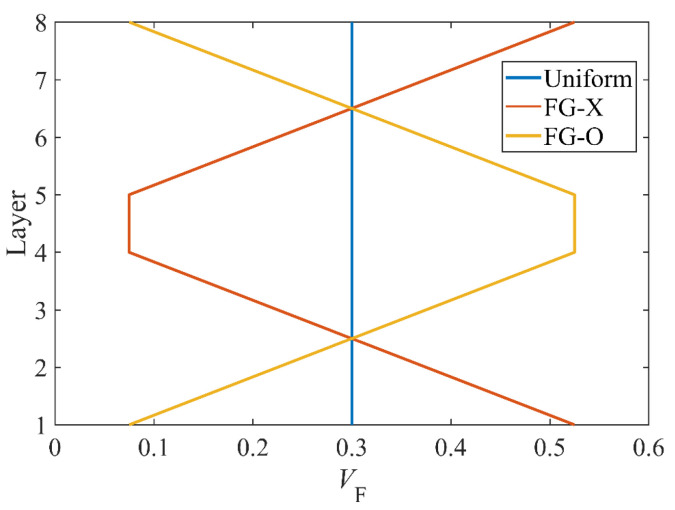
Volume fraction in each layer; *N*_L_ = 8.

Figure 4 plots the volume fraction in each layer (*N*_L_ = 8). It shows that uniform distribution (UD) is a special case of an orthotropic homogeneous plate; FG-X has a CF volume fraction that increases at the top and bottom layers of the plate; and FG-O has a CF volume fraction that increases at the central layer of the plate. Furthermore, all the distribution patterns of plates are symmetric according to the mid-surface of the plate.

The thermal expansion coefficients in the longitudinal and transverse directions are stated as:

(9) $$\alpha_{11}=\frac{V_{F}E_{11}^{F}\alpha_{11}^{F}+\left(1-V_{F}\right)E^{M}\alpha^{M}}{V_{F}E_{11}^{F}+\left(1-V_{F}\right)E^{M}}$$

(10) $$\alpha_{22}=V_{F}\left(1+v_{12}^{F}\right)\alpha_{22}^{F}+\left(1-V_{F}\right)\left(1+v^{M}\right)\alpha^{M}-v_{12}\alpha_{11}$$

where $\alpha_{11}^{F}$ and $\alpha_{22}^{F}$ are the thermal expansion coefficients of the fiber, and $\alpha^{M}$ is the thermal expansion coefficient of the matrix. Therefore, the thermal expansion coefficients of CFRP are functions of the volume fraction.

The laminated plate is exposed to the thermal environment. The temperature distributions are assumed through the thickness of the plate by three types of distribution; however, the present paper only concerns uniform distribution:

(11) $$T\left(z\right)=T_{0}+\Delta T\;$$

where Δ*T* is the temperature rise from the reference temperature, at which there are no thermal strains. *T*_0_ indicate the reference temperature.

We assume that the material properties of the matrix *E*^M^ and *α*^M^ are functions of the temperature; hence, all the effective material properties of CFRP are functions of the temperature and CF volume fraction.

## 3. Theoretical Formulation

### 3.1. Displacement Field Model

Based on the classical laminate plate theory [43], the displacement field of the laminated plate theory can be expressed as:

(12) $$u\left(x,y,z,t\right)=u_{0}\left(x,y,t\right)-z\frac{\partial w_{0}}{\partial x}$$

(13) $$v\left(x,y,z,t\right)=v_{0}\left(x,y,t\right)-z\frac{\partial w_{0}}{\partial y}$$

(14) $$w\left(x,y,z,t\right)=w_{0}\left(x,y,t\right)$$

where (*u*_0_, *v*_0_, *w*_0_) are the displacement components along each coordinate direction of a point on the midplane (*z* = 0). The displacement field implies that straight lines normal to the *xy*-plane before deformation remain straight and normal to the mid-surface after deformation. The strain-displacement relations are as follows:

(15) $$\left[\begin{array}{c} \varepsilon_{x}\\ \varepsilon_{y}\\ \gamma_{xy} \end{array}\right]=\left[\begin{array}{c} \varepsilon_{x}^{o}\\ \varepsilon_{y}^{o}\\ \gamma_{xy}^{o} \end{array}\right]+z\left[\begin{array}{c} \kappa_{x}\\ \kappa_{y}\\ \kappa_{xy} \end{array}\right]$$

where $\varepsilon_{x}^{o},\;\varepsilon_{y}^{o},\;\gamma_{xy}^{o}$ are the mid-plane strains:

(16) $$\left[\begin{array}{c} \kappa_{x}\\ \kappa_{y}\\ \kappa_{xy} \end{array}\right]=\left[\begin{array}{c} -\frac{\partial^{2}w_{0}}{\partial x^{2}}\\ -\frac{\partial^{2}w_{0}}{\partial y^{2}}\\ -2\frac{\partial^{2}w_{0}}{\partial x\partial y} \end{array}\right]$$

Based on the von Kármán anisotropic plate equations for large deflections [44]:

(17) $$\begin{array}{l} \varepsilon_{x}^{o}=\frac{\partial u}{\partial x}+\frac{1}{2}{\left(\frac{\partial w}{\partial x}\right)}^{2}\\ \varepsilon_{y}^{o}=\frac{\partial v}{\partial y}+\frac{1}{2}{\left(\frac{\partial w}{\partial y}\right)}^{2}\\ \gamma_{xy}^{o}=\frac{\partial u}{\partial y}+\frac{\partial v}{\partial x}+\left(\frac{\partial w}{\partial x}\right)\left(\frac{\partial w}{\partial y}\right) \end{array}$$

In thermal environments, the constitutive relations are written as:

(18) $${\left[\begin{array}{c} \sigma_{x}\\ \sigma_{y}\\ \tau_{xy} \end{array}\right]}^{\left(k\right)}={\left[\begin{array}{ccc} {\bar{Q}}_{11} & {\bar{Q}}_{12} & {\bar{Q}}_{16}\\ {\bar{Q}}_{12} & {\bar{Q}}_{22} & {\bar{Q}}_{26}\\ {\bar{Q}}_{16} & {\bar{Q}}_{26} & {\bar{Q}}_{66} \end{array}\right]}^{\left(k\right)}\left(\left[\begin{array}{c} \varepsilon_{x}\\ \varepsilon_{y}\\ \gamma_{xy} \end{array}\right]-{\left[\begin{array}{c} {\bar{\alpha }}_{x}\\ {\bar{\alpha }}_{y}\\ {\bar{\alpha }}_{xy} \end{array}\right]}^{\left(k\right)}\Delta T\right)$$

where the transformed coefficients of stiffness are as follows [43]:

(19) $$\begin{array}{l} {\bar{Q}}_{11}=Q_{11}\cos^{4}\theta +2\left(Q_{12}+2Q_{66}\right)\cos^{2}\theta \sin^{2}\theta +Q_{22}\sin^{4}\theta \\ {\bar{Q}}_{22}=Q_{11}\sin^{4}\theta +2\left(Q_{12}+2Q_{66}\right)\cos^{2}\theta \sin^{2}\theta +Q_{22}\cos^{4}\theta \\ {\bar{Q}}_{12}=\left(Q_{11}+Q_{22}-4Q_{66}\right)\cos^{2}\theta \sin^{2}\theta +Q_{12}\left(\cos^{4}\theta +\sin^{4}\theta \right)\\ {\bar{Q}}_{16}=\left(Q_{11}-Q_{12}-2Q_{66}\right)\cos^{3}\theta \sin\theta +\left(Q_{12}-Q_{22}+2Q_{66}\right)\cos\theta \sin^{3}\theta \\ {\bar{Q}}_{26}=\left(Q_{11}-Q_{12}-2Q_{66}\right)\cos\theta \sin^{3}\theta +\left(Q_{12}-Q_{22}+2Q_{66}\right)\cos^{3}\theta \sin\theta \\ {\bar{Q}}_{66}=\left(Q_{11}+Q_{22}-2Q_{12}-2Q_{66}\right)\cos^{2}\theta \sin^{2}\theta +Q_{66}\left(\cos^{4}\theta +\sin^{4}\theta \right) \end{array}$$

with

(20) $$Q_{11}=\frac{E_{11}}{1-v_{12}v_{21}},Q_{22}=\frac{E_{22}}{1-v_{12}v_{21}},Q_{12}=\frac{v_{12}E_{22}}{1-v_{12}v_{21}},Q_{66}=G_{12}$$

The transformed coefficients of thermal expansion are denoted as:

(21) $$\begin{array}{l} {\bar{\alpha }}_{x}=\alpha_{11}\cos^{2}\theta +\alpha_{22}\sin^{2}\theta \\ {\bar{\alpha }}_{y}=\alpha_{11}\sin^{2}\theta +\alpha_{22}\cos^{2}\theta \\ {\bar{\alpha }}_{xy}=2\left(\alpha_{11}-\alpha_{22}\right)\sin\theta \cos\theta \end{array}$$

### 3.2. Buckling Equations

The variation of the strain energy of the laminated plate is calculated in Equation (22).

(22) $$\begin{array}{l} \delta U=\int_{V}\left[\sigma_{x}^{\left(n\right)}\delta \varepsilon_{x}^{\left(n\right)}+\sigma_{y}^{\left(n\right)}\delta \varepsilon_{y}^{\left(n\right)}+\tau_{xy}^{\left(n\right)}\delta \gamma_{xy}^{\left(n\right)}\right]dV\\ =\int_{\Omega }\left(N_{x}\delta \varepsilon_{x}^{\left(0\right)}+M_{x}^{}\delta k_{x}^{}+N_{y}\delta \varepsilon_{y}^{\left(0\right)}+M_{y}^{}\delta k_{y}^{}+N_{xy}\delta \gamma_{xy}^{\left(0\right)}+M_{xy}^{}\delta k_{xy}^{}\right)d\Omega \end{array}$$

The work performed by external forces can be defined as:

(23) $$W=-\frac{1}{2}\iint \left[N_{x}^{0}{\left(\frac{\partial w}{\partial x}\right)}^{2}+N_{y}^{0}{\left(\frac{\partial w}{\partial y}\right)}^{2}+2N_{xy}^{0}\frac{\partial w}{\partial x}\frac{\partial w}{\partial y}\right]d\Omega$$

where $N_{x}^{0}$, $N_{y}^{0}$ and $N_{xy}^{0}$ stand for in-plane compression loads per unit length.

The principle of virtual work for the present problem can be expressed as:

(24) $$\int_{\Omega }\left(N_{x}\delta \varepsilon_{x}^{\left(0\right)}+M_{x}^{}\delta k_{x}^{}+N_{y}\delta \varepsilon_{y}^{\left(0\right)}+M_{y}^{}\delta k_{y}^{}+N_{xy}\delta \gamma_{xy}^{\left(0\right)}+M_{xy}^{}\delta k_{xy}^{}\right)d\Omega -\int_{\Omega }\bar{N}\delta wd\Omega =0$$

where

(25) $$\bar{N}=\left(N_{x}^{0}\frac{\partial^{2}w}{\partial x^{2}}+N_{y}^{0}\frac{\partial^{2}w}{\partial y^{2}}+2N_{xy}^{0}\frac{\partial^{2}w}{\partial x\partial y}\right)$$

where the force resultants are as follows:

(26) $$\left[N_{j},M_{j}\right]=\sum_{k=1}^{n}\int_{z_{k}}^{z_{k+1}}\sigma_{j}^{\left(k\right)}\left[1,z\right]dz,\left(j=x,y,xy\right)$$

In a thermal environment, the above force resultants can be expressed in terms of strains as follows:

(27) $$\begin{array}{l} \left[\begin{array}{c} N_{x}\\ N_{y}\\ N_{xy} \end{array}\right]=\left[\begin{array}{ccc} A_{11} & A_{12} & A_{16}\\ A_{12} & A_{22} & A_{26}\\ A_{16} & A_{26} & A_{66} \end{array}\right]\left[\begin{array}{c} \varepsilon_{x}^{o}\\ \varepsilon_{y}^{o}\\ \gamma_{xy}^{o} \end{array}\right]+\left[\begin{array}{ccc} B_{11} & B_{12} & B_{16}\\ B_{12} & B_{22} & B_{26}\\ B_{16} & B_{26} & B_{66} \end{array}\right]\left[\begin{array}{c} \kappa_{x}\\ \kappa_{y}\\ \kappa_{xy} \end{array}\right]-\left[\begin{array}{c} N_{x}^{T}\\ N_{y}^{T}\\ N_{xy}^{T} \end{array}\right] \end{array}$$

(28) $$\left[\begin{array}{c} M_{x}\\ M_{y}\\ M_{xy} \end{array}\right]=\left[\begin{array}{ccc} B_{11} & B_{12} & B_{16}\\ B_{12} & B_{22} & B_{26}\\ B_{16} & B_{26} & B_{66} \end{array}\right]\left[\begin{array}{c} \varepsilon_{x}^{o}\\ \varepsilon_{y}^{o}\\ \gamma_{xy}^{o} \end{array}\right]+\left[\begin{array}{ccc} D_{11} & D_{12} & D_{16}\\ D_{12} & D_{22} & D_{26}\\ D_{16} & D_{26} & D_{66} \end{array}\right]\left[\begin{array}{c} \kappa_{x}\\ \kappa_{y}\\ \kappa_{xy} \end{array}\right]-\left[\begin{array}{c} M_{x}^{T}\\ M_{y}^{T}\\ M_{xy}^{T} \end{array}\right]$$

where the stiffness matrices are as follows:

(29) $$\left[A_{ij},B_{ij},D_{ij}\right]=\sum_{k=1}^{N}\int_{z_{k}}^{z_{k+1}}{\bar{Q}}_{ij}^{\left(k\right)}\left[1,z,z^{2}\right]dz,\;\left(i,j=1,2,6\right)$$

where the thermal stress and moment are as follows:

(30) $$\left[\begin{array}{c} N_{x}^{T}\\ N_{y}^{T}\\ N_{xy}^{T} \end{array}\right]=\int_{-\frac{t}{2}}^{\frac{t}{2}}{\left[\begin{array}{ccc} {\bar{Q}}_{11} & {\bar{Q}}_{12} & {\bar{Q}}_{16}\\ {\bar{Q}}_{12} & {\bar{Q}}_{22} & {\bar{Q}}_{26}\\ {\bar{Q}}_{16} & {\bar{Q}}_{26} & {\bar{Q}}_{66} \end{array}\right]}^{\left(k\right)}{\left[\begin{array}{c} {\bar{\alpha }}_{x}\\ {\bar{\alpha }}_{y}\\ {\bar{\alpha }}_{xy} \end{array}\right]}^{\left(k\right)}\Delta T^{\left(k\right)}dz$$

(31) $$\left[\begin{array}{c} M_{x}^{T}\\ M_{y}^{T}\\ M_{xy}^{T} \end{array}\right]=\int_{-\frac{t}{2}}^{\frac{t}{2}}{\left[\begin{array}{ccc} {\bar{Q}}_{11} & {\bar{Q}}_{12} & {\bar{Q}}_{16}\\ {\bar{Q}}_{12} & {\bar{Q}}_{22} & {\bar{Q}}_{26}\\ {\bar{Q}}_{16} & {\bar{Q}}_{26} & {\bar{Q}}_{66} \end{array}\right]}^{\left(k\right)}{\left[\begin{array}{c} {\bar{\alpha }}_{x}\\ {\bar{\alpha }}_{y}\\ {\bar{\alpha }}_{xy} \end{array}\right]}^{\left(k\right)}\Delta T^{\left(k\right)}zdz$$

The stability equations of the plate may be derived by the adjacent equilibrium criterion [45]. Assume that the equilibrium state of the plate under mechanical and thermal loads is defined in terms of the displacement components $\left(u_{0}^{0},\;v_{0}^{0},\;w_{0}^{0}\right)$. The displacement components of a neighboring stable state differ by $\left(u_{0}^{1},\;v_{0}^{1},\;w_{0}^{1}\right)$ with respect to the equilibrium position. Thus, the total displacements of a neighboring state are as follows:

(32) $$u_{0}=u_{0}^{0}+u_{0}^{1},\;v_{0}=v_{0}^{0}+v_{0}^{1},\;w_{0}=w_{0}^{0}+w_{0}^{1}$$

Substituting Equations (15), (27) and (32) into Equation (24), integrating the displacement gradients by parts and then setting the coefficients $\delta u_{0}^{1},\delta v_{0}^{1}$ and $\delta w_{}^{1}$ to zero separately, the governing stability equations are calculated as in Equation (33).

(33) $$\frac{\partial^{2}M_{x}^{1}}{\partial x^{2}}+2\frac{\partial^{2}M_{xy}^{1}}{\partial x\partial y}+\frac{\partial^{2}M_{y}^{1}}{\partial y^{2}}+\bar{N}+{\bar{N}}^{T}=0$$

in which

(34) $${\bar{N}}^{T}=-N_{x}^{T}\frac{\partial^{2}w_{0}^{1}}{\partial x^{2}}-N_{y}^{T}\frac{\partial^{2}w_{0}^{1}}{\partial y^{2}}-2N_{xy}^{T}\frac{\partial^{2}w_{0}^{1}}{\partial x\partial y}$$

In the present paper, the laminate is symmetric, so the bending–stretching matrix [B] = 0. Moreover, we assume that *D*_16_ and *D*_26_ in the bending matrix [D] are zero, so Equation (33) can be expressed as Equation (35).

(35) $$-D_{11}\frac{\partial^{4}w_{0}^{1}}{\partial x^{4}}-2\left(D_{12}+2D_{66}\right)\frac{\partial^{4}w_{0}^{1}}{\partial x^{2}\partial y^{2}}-D_{22}\frac{\partial^{4}w_{0}^{1}}{\partial y^{4}}+\left(N_{x}^{0}-N_{x}^{T}\right)\frac{\partial^{2}w_{0}^{1}}{\partial x^{2}}+\left(N_{y}^{0}-N_{y}^{T}\right)\frac{\partial^{2}w_{0}^{1}}{\partial y^{2}}+2\left(N_{xy}^{0}-N_{xy}^{T}\right)\frac{\partial^{2}w_{0}^{1}}{\partial x\partial y}=0$$

The boundary conditions are calculated in Equation (36).

(36) $$\begin{array}{l} w=M_{x}=0\;x=0\;x=a\\ w=M_{y}=0\;y=0\;y=b \end{array}$$

An expression for $w_{0}^{1}$ that satisfies all the boundary conditions takes the form of the following double trigonometric series.

(37) $$w_{0}^{1}=\sum \sum A_{mn}\sin\frac{m\pi x}{a}\sin\frac{n\pi y}{b}$$

where *m* and *n* are the number of half-waves in the *x* direction and *y* direction, respectively, and *A_mn_* is the coefficients.

Therefore, for the special orthotropic and symmetric laminated plates, Equation (35) can be expressed as follows.

(38) $$\pi^{2}A_{mn}\left[D_{11}m^{4}+2(D_{16}+2D_{66})m^{2}n^{2}{\left(AR\right)}^{2}+D_{22}n^{4}{\left(AR\right)}^{4}\right]=-A_{mn}a^{2}\left[{\bar{N}}_{x}m^{2}+{\bar{N}}_{y}n^{2}{\left(AR\right)}^{2}\right]$$

Since *A_mn_ ≠ 0*

(39) $$N_{0}=\frac{\pi^{2}\left[D_{11}m^{4}+2\left(D_{12}+2D_{66}\right)m^{2}n^{2}{\left(k_{1}\right)}^{2}+D_{22}n^{4}{\left(k_{1}\right)}^{4}\right]}{a^{2}\left[m^{2}+k_{0}n^{2}{\left(k_{1}\right)}^{2}\right]}-N_{x}^{T}$$

where $k_{0}=N_{y}^{0}/N_{x}^{0}$, *k*_1_ *= a/b*, $N_{0}=-N_{x}^{0}$, the stiffness matric *D_ij_* (*i*, *j* = 1,2,6) and the thermal stress $N_{x}^{T}$ are functions of the temperature and CF volume fraction.

Clearly, for each pair of *m* and *n*, there is a unique *N*_0_, according to Equation (39). The critical buckling load is the smallest value of all *N*_0_ *= N*_0_(*m*, *n*), and it can be obtained with Equation (40).

(40) $$N_{\mathrm{cr}}=\underset{1\leq m,n\leq \infty }{\min}\left\{N_{0}\left(m,n\right)\right\}$$

Non-dimensional critical buckling load can be expressed as Equation (41).

(41) $$\gamma_{\mathrm{cr}}=\frac{N_{\mathrm{cr}}b^{2}}{\pi^{2}D_{22}}$$

## 4. Result and Discussion

### 4.1. Validation

Here, we need a paragraph to describe the validation in detail. A comparison of the present results with published results and finite element analysis is given in Table 1 and Table 2. As a verification example, the buckling load of a simply supported laminated plate is calculated and compared. In Table 1, the material properties of the laminated plate (*a* = *b*) are considered as in [43]: *G*_12_ = *G*_13_ = 0.5 *E*_2_, *v*_12_ = 0.25. The non-dimensional buckling loads are $\lambda_{\mathrm{cr}}=N_{\mathrm{cr}}\left(b^{2}/\pi^{2}D_{22}\right)$. The finite element analysis is performed using Workbench 19.0 software. The simply supported plates under uniform compression and biaxial compression are modeled in Workbench software using the four-node element with six degrees of freedom at each node. In Table 2, the material properties of the laminated plate (*a* = *b*) are adopted as in [40]: aluminum—*E*_m_ = 70 GPa, *v* = 0.3; alumina—*E*_c_ = 380 GPa, *v* = 0.3. The non-dimensional buckling loads are $\lambda_{\mathrm{cr}}{=N_{cr}b}^{2}/E_{c}h^{3}$. It can be seen that the present results for an anisotropic and isotropic plate are in good agreement with the analytical results obtained in Equation (39).

### 4.2. Parametric Studies

In this section, a parametric study is carried out to reveal the buckling properties of the simply supported laminated plates. The effects of the CF distribution pattern and volume fraction, total number of layers, temperature, geometrical dimension and lamination angle are investigated. The material properties of the CFRP composite laminated plates are listed in Table 3. Unless otherwise specified, the geometry of the laminated plate is defined as *a* = 80 mm.

**Figure 5:**
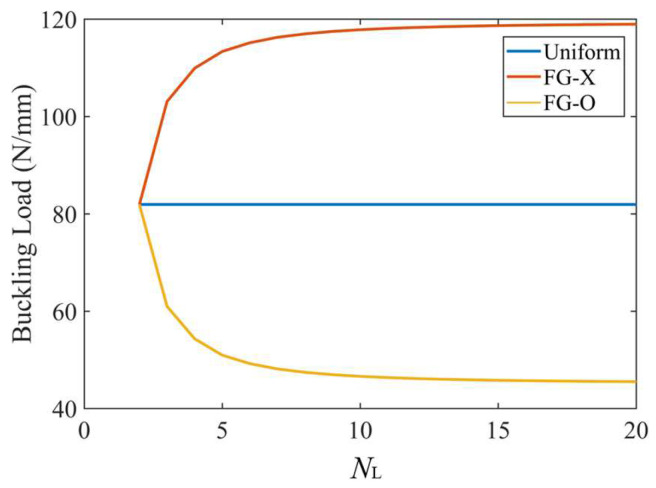
Effect of total number of layers *N*_L_ on the critical buckling load for the UD, FG-O and FG-X CFRP plates; *a* = 80 mm, *k*_0_ = 0, *k*_1_ = 1, *k*_2_ = 50, [45°]*_N_*_L_.

Figure 5 displays the effect of the total number of layers, *N*_L_, on the critical buckling load for the UD, FG-O and FG-X CFRP laminated plates. We can see that the buckling load of the UD distribution pattern is not affected by the number of layers. As the total number of layers increases up to 10–15, the buckling load increases within an increase of the total number of layers for the FG-X distribution pattern, but becomes lower for the FG-O distribution pattern, and then almost unchanged when *N*_L_ ≥ 10–15. The multilayer structure with 10~15 layers stacked up would be accurate enough to approximate the desired continuous and smooth through-thickness change in the CF distribution. This is consistent with the results in Ref. [38]. In order to simplify the parameter study, in the present paper, the total number of layers of the laminated plate is defined as *N*_L_ = 8 when the buckling load of the FG-X distribution pattern is 42% higher than that of the UD.

The effect of the lamination scheme is demonstrated in Table 4, where a comparison is also made between the buckling loads obtained via analytical and finite element analyses. In the present paper, the method is applied to parallel-fiber or cross-ply plates, but the results of this study are in close agreement with the finite element analysis in the random-twenty laminate schemes; we conclude that the method can accurately predict the buckling load of the plates discussed in this paper. We also find that 45° and −45° in the same layer have the same buckling load for three distribution patterns. Meanwhile, 0° and 90° in the same layer have the same buckling load for uniform patterns. The result confirms that the [−45/45/90/0]_s_, [−45/45/0/90]_s_, [45/−45/90/0]_s_ and [45/−45/0/90]_s_ plates have the highest buckling load among the twenty laminate arrangements. Unless otherwise specified, in the present paper, the eight-layer symmetric lamination scheme [−45/45/90/0]_s_ is considered for the analysis, and the buckling load of the FG-X distribution pattern is 47% higher than that of the UD.

#### 4.2.1. Effect of Carbon Fiber Volume Fraction

The effect of the volume fraction $V_{F}^{*}$ on the buckling loads for UD, FG-X and FG-O plates under uniaxial and equal biaxial compressions is presented in Table 5. An increase of the buckling load is found when the CF average volume fraction increases in our considered ranges. The buckling load increases by more than 40% when the average volume fraction increases from 0.05 to 0.1, but the slope of the buckling load decreases, in which, the buckling load slope is calculated as $\left|N_{cr}^{}\left(V_{F}^{*}\right)-N_{cr}^{}\left(V_{F}^{*}-0.05\right)\right|/N_{cr}^{}\left(V_{F}^{*}-0.05\right)$, where $N_{cr}^{}\left(V_{F}^{*}\right)$ and $N_{cr}^{}\left(V_{F}^{*}-0.05\right)$ are the buckling loads when the average volume fraction is $V_{F}^{*}$ and $V_{F}^{*}-0.05$. The slope goes down to less than 20% when the average volume fraction is 0.3. It has also been demonstrated that the CF distribution pattern plays a significant role in the buckling load of the plate. FG-X has a larger buckling load when the average volume fraction of CF is the same. For this reason, it can be concluded that the stiffness of the plate can be improved in the range where the average volume fraction increases. At the same time, distributing more CFs close to the top and bottom surfaces is a more efficient way to improve the plate stiffness.

#### 4.2.2. Effect of Thermal Environment

**Figure 6:**
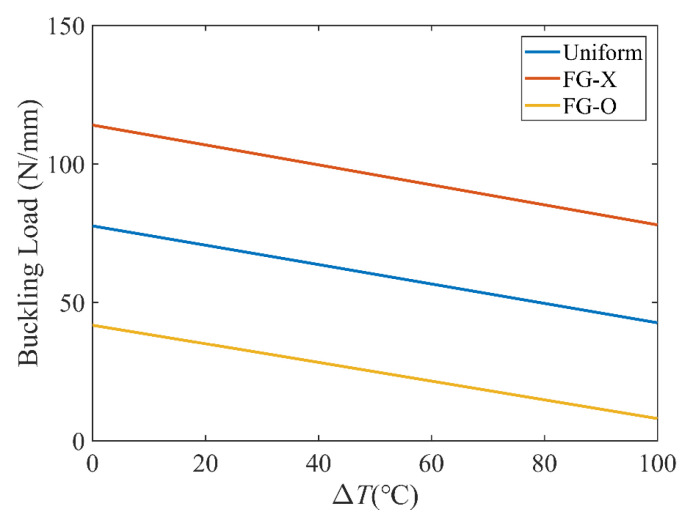
Effect of temperature on buckling load; *a* = 80 mm, *k*_0_ =0, *k*_1_ = 1, *k*_2_ = 50.

**Figure 7:**
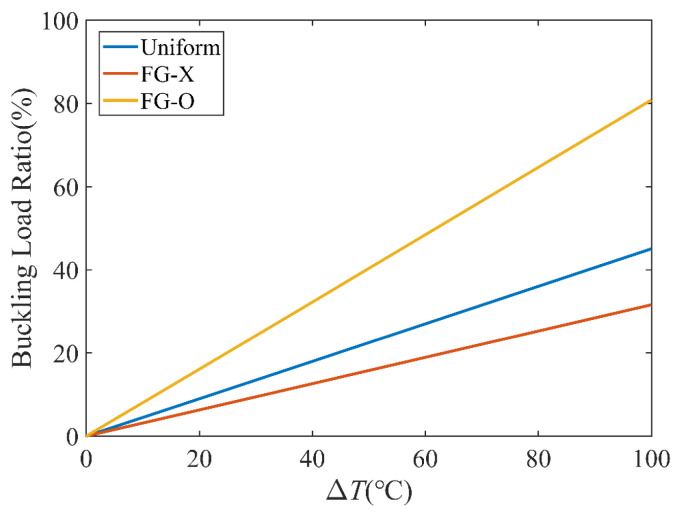
Effect of temperature on buckling load ratio; *a* = 80 mm, *k*_0_ = 0, *k*_1_ = 1, *k*_2_ = 50.

Figure 6 investigates the effect of the temperature on the buckling load. It can be seen that the buckling load parameters decrease as the temperature increases, because with an increase of the temperature, the elastic modulus of CFRC reduces and the stiffness of the laminated plates reduce since the material properties of the matrix and CFs are assumed to be temperature-dependent, and the resultant stress is reduced by the thermal stress and the momentum produced by the thermal effects. For the three distribution patterns, the lowest and highest buckling loads correspond to FG-O and FG-X. Moreover, the effect of the temperature on the buckling load ratio of the FG CFRC plate is investigated in Figure 7. The buckling load ratio is calculated as $\left|N_{cr}^{T}-N_{cr}^{T_{0}}\right|/N_{cr}^{T_{0}}$, where $N_{cr}^{T_{0}}$ and $N_{cr}^{T}$ are the buckling loads at the reference and actual temperatures. FG-O has a much larger buckling load ratio than that of FG-X.

#### 4.2.3. Effect of Geometrical Dimension of Plate

**Figure 8:**
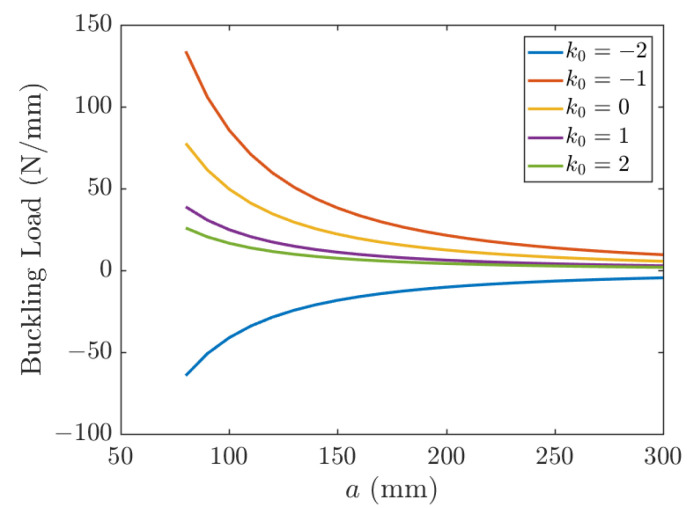
Effect of length on the buckling of a UD CFRP; *k*_1_ = 1, *k*_2_ = 50.

The effect of length on the buckling of a UD CFRP is depicted in Figure 8. Five different biaxial loading ratios, *k*_0_ = −2, *k*_0_ = −1, *k*_0_ = 0, *k*_0_ = 1, *k*_0_ = 2, are considered. Positive values of *k*_0_ mean that the sign of $N_{x}^{0}$ is the same as $N_{y}^{0}$ and since *N*_cr_ > 0 implies biaxial compression. Then, negative values of *k*_0_ mean that $N_{x}^{0}$ is opposite to $N_{y}^{0}$. When *N*_cr_ > 0, buckling occurs in the *x* direction; otherwise, buckling occurs in the y direction. As is seen in Figure 8, a tensile load *N*_y_ (*k*_0_ < 0) tends to stabilize the plate and increase its buckling load. A compressive load *N*_y_ (*k*_0_ > 0) tends to precipitate the buckling earlier (plate is pressed from both x and y directions) and decreases the buckling load. The case of *k*_0_ = 0 corresponds to uniaxial compression. The buckling load is shown in Figure 8 as a function of the plate size and different load ratio *k*_0_.

**Figure 9:**
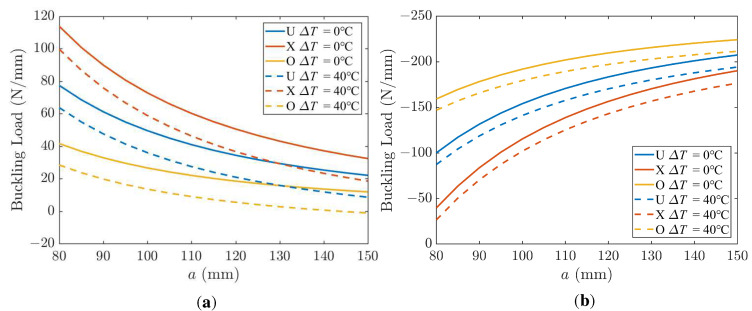
Effect of length on buckling of a UD CFRP (*k*_2_ = 50); (**a**) *k*_0_ = 0, *k*_1_ = 1; (**b**) *k*_0_ = −1, *k*_1_ = 2.

Figure 9 illustrates the effect of length and temperature on the buckling load for the three distribution patterns. Increasing the plate size decreases the buckling load for all three distribution patterns, and the buckling load of FG-X reduces the fastest with the increase of length.

**Figure 10:**
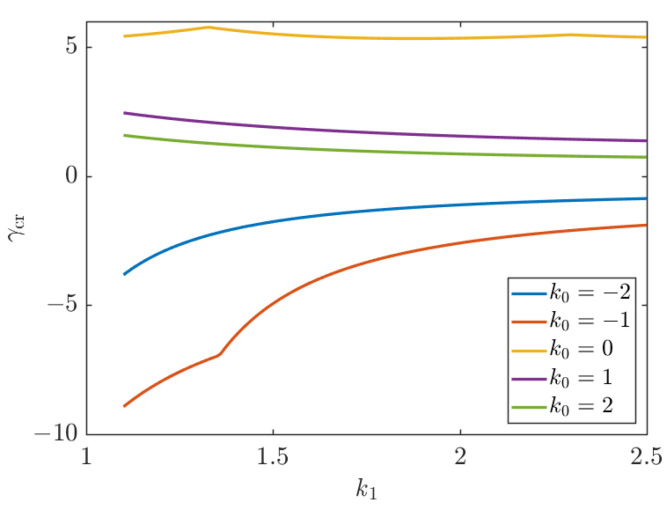
Effect of aspect ratio on non-dimensional critical buckling load; *k*_2_ = 50.

Figure 10 shows the effect of the aspect ratio on the non-dimensional critical buckling load. It is worth noting that for CFRC plates under uniaxial compression, as shown in Figure 10, the variation of the aspect ratio of the plate has a very small effect on the non-dimensional buckling load parameter. Meanwhile, the biaxial non-dimensional buckling load decreases with an increasing aspect ratio of the plate.

**Figure 11:**
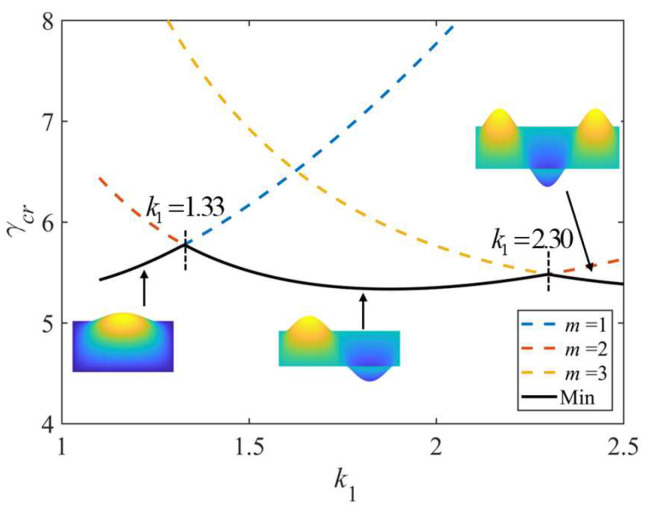
Effect of aspect ratio and the number of half-waves *m* on non-dimensional critical buckling load; *k*_0_ = 0, *k*_2_ = 50.

As shown in Figure 11, the effect of the aspect ratio and the number of half-waves *m* on the non-dimensional critical buckling load (*k*_0_ = 0) is such that when the number of half-waves *m* on the buckling load takes on different values, the minimum value is taken as the buckling load. As the aspect ratio increases, the value of *m* in the buckling load direction increases. Points (*k*_1_ = 1.33, *k*_1_ = 2.30) of the inflection of the curves corresponding to successive *m* values indicate that the plate may experience buckling in either of the two modes (differing by one half-wave) and have the same buckling load. In the following, there are inflection points on the non-dimensional critical buckling load curve due to the change of the number of half-waves *m*.

**Figure 12:**
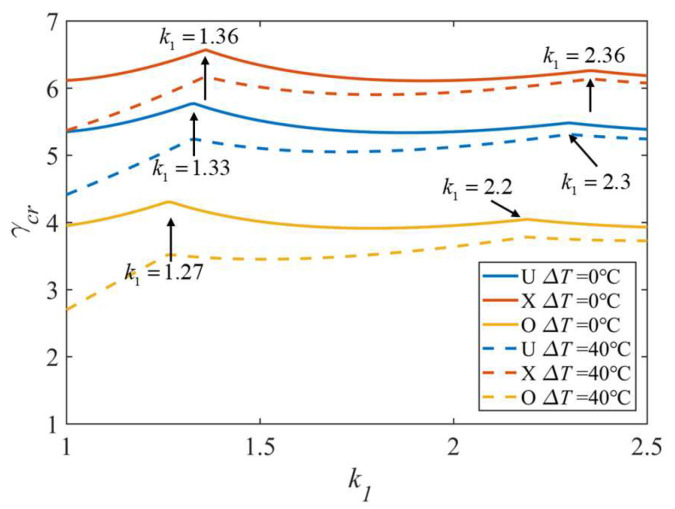
Effect of aspect ratio and temperature difference for three distribution patterns; *k*_0_ = 0, *k*_2_ = 50.

Figure 12 illustrates the effect of the aspect ratio and temperature for the three distribution patterns. In each case, three types of CFs distributions are taken into consideration. There are two inflection points for each curve, and the value of *k*_1_ at the inflection point is larger for FG-X than for FG-O and uniform. Table 6 gives a detailed comparison of some data in Figure 12. The effect of the temperature difference for FG-O is greater than those for the FG-X and uniform; the temperature difference results in a 31.65% reduction in the FG-O non-dimensional critical buckling load when *k*_1_ = 1, and the reduction goes down to 5.09% when *k*_1_ = 2.5. The results indicate that the temperature change reduces the non-dimensional critical buckling load.

**Figure 13:**
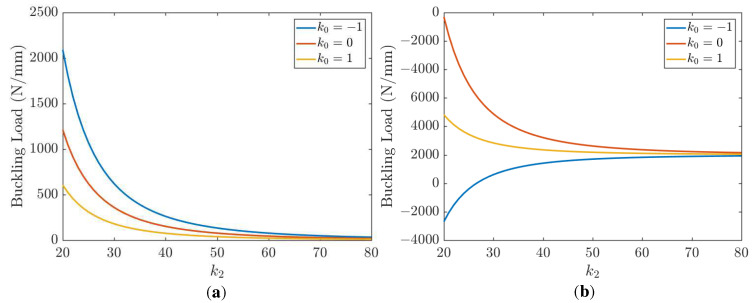
Effect of length-to-thickness ratio *k*_2_: (**a**) *k*_1_ = 1; (**b**) *k*_1_ = 2.

Figure 13 shows the effect of the length-to-thickness ratio *k*_2_ in aspect ratio *k*_1_ = 1 and *k*_1_ = 2, respectively. Compared with (a) and (b), it can be found that the buckling load rapidly decreases with the increase of the length-to-thickness ratio *k*_2_. This is because the stiffness depends on the integral of *z*^2^ along the thickness, and the stiffness is lowered by the reduction of the thickness. The results also show that the effect of the distribution of CFs becomes weaker for moderately thick CFRC plates.

**Figure 14:**
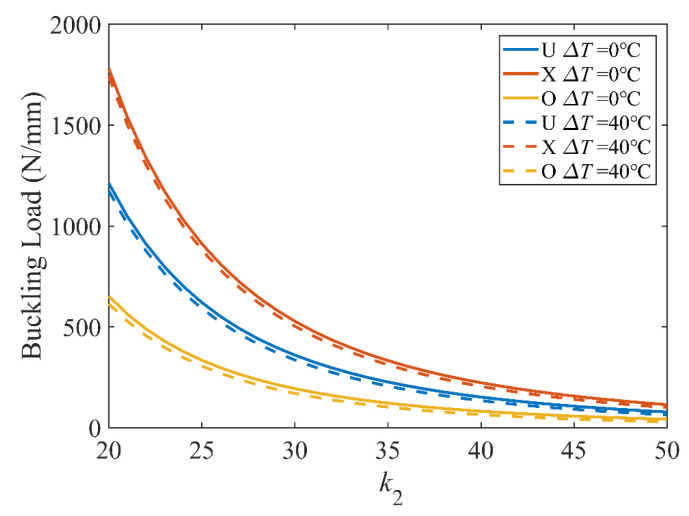
Effect of length-to-thickness ratio *k*_2_ and temperature difference for three distribution patterns; *k*_0_ = 0, *k*_1_ = 1.

Figure 14 reveals the effect of the length-to-thickness ratio *k*_2_ and the temperature difference for the three distribution patterns. Similar to the conclusions above, the buckling load decreases with increasing temperature, but the effect of thickness dominates.

#### 4.2.4. Effect of Lamination Angle

**Figure 15:**
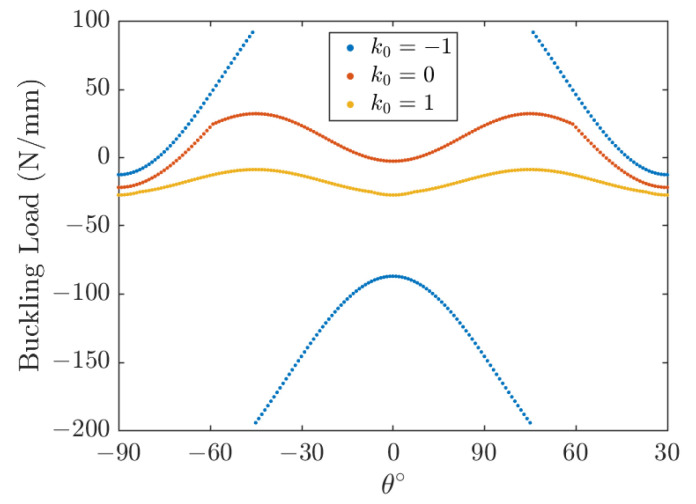
Effect of lamination angle on buckling load; *k*_1_ = 1, *k*_2_ = 50, [*θ*].

**Figure 16:**
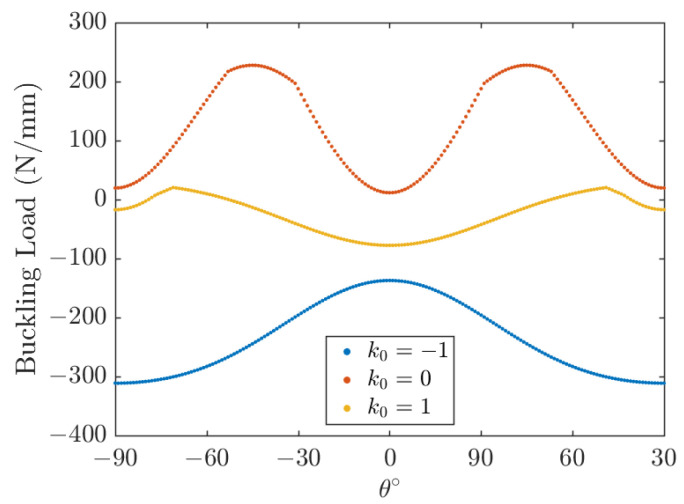
Effect of lamination angle on buckling load; *k*_1_ = 2, *k*_2_ = 50, [*θ*].

The effect of the fiber angle is shown in Figure 15 and Figure 16 for square and rectangular plates, respectively, for the length-to-thickness ratio *k*_2_ = 50. The plots shown in Figure 15 and Figure 16 are symmetric about *θ* = 0°. It is also demonstrated that the aspect ratio *k*_1_ plays a significant role in the optimal lamination angle of the plate. In Figure 15, it is worth noting that the buckling load increases with the increase of the lamination angle *θ* and decreases very quickly with a further increase of the lamination angle *θ*. Negative values of *k*_0_ (*k*_0_ = −1) mean that $N_{x}^{0}$ is opposite to $N_{y}^{0}$, and when *θ* = ±45°, the buckling direction changes from the *x* direction to the *y* direction. In conclusion, the buckling load is the maximum at *θ* = 45° for a square plate under three types of load and a rectangular plate under uniaxial compression (*k*_0_ = 0); the rectangle plate buckling load under biaxial compression (*k*_0_ = 1) and opposite load (*k*_0_ = −1) is the maximum in *θ* = 70° and *θ* = 90°, respectively. The angle of lamination that maximizes the buckling load is related to the aspect ratio of the plate.

**Figure 17:**
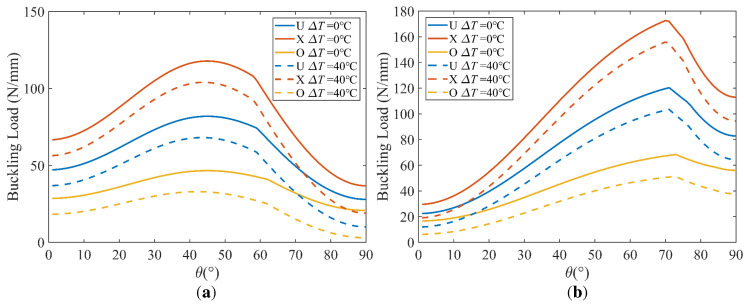
Effect of lamination angle and temperature difference for three distribution patterns: (**a**) *k*_0_ = 0, *k*_1_ = 1, *k*_2_ = 50; (**b**) *k*_0_ = 1, *k*_1_ = 2, *k*_2_ = 50.

Figure 17 presents the effect of the lamination angle and temperature for the three distribution patterns. Compared with Figure 15 and Figure 16, we find that the angle that maximizes the buckling load is approximately the same for the three distribution patterns, and it is not affected by the temperature.

**Figure 18:**
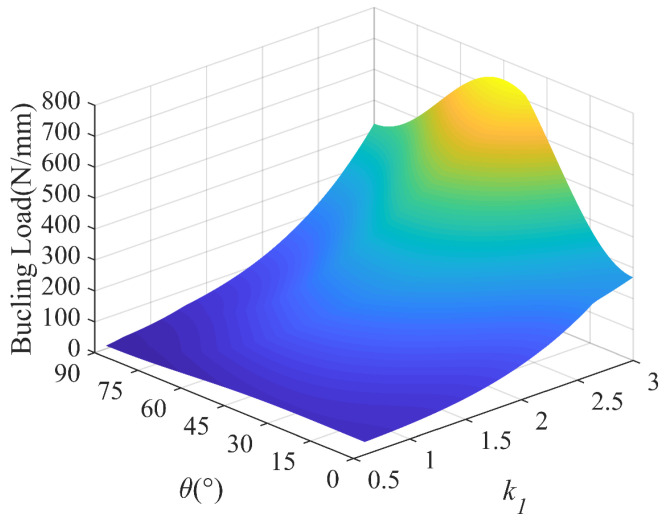
Buckling load with lamination angle and aspect ratio; *k*_0_ = 0, *k*_2_ = 50.

**Figure 19:**
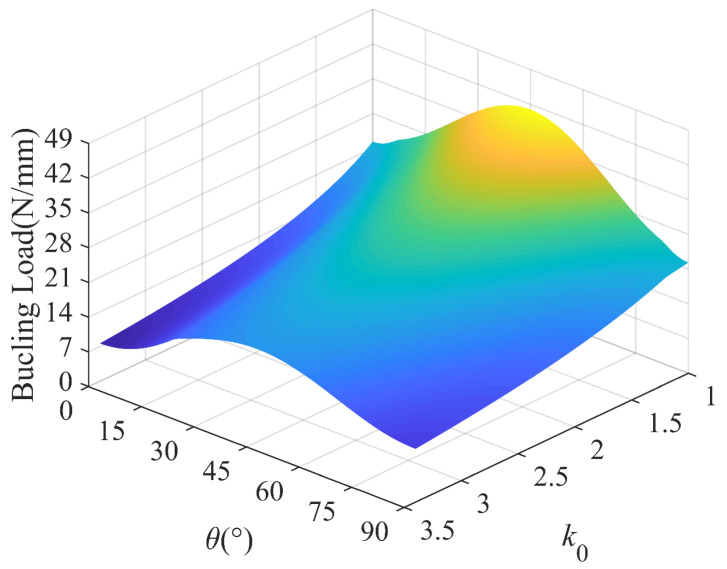
Buckling load with lamination angle and compression load ratio; *k*_1_ = 1, *k*_2_ = 50.

**Figure 20:**
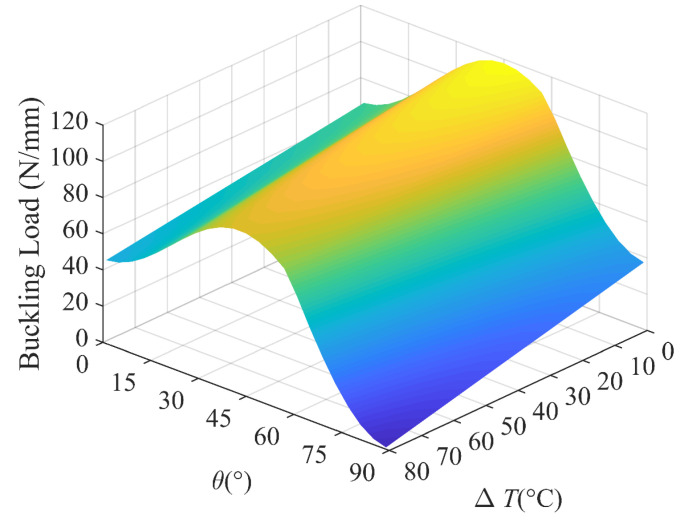
Buckling load with lamination angle and temperature difference; *k*_0_ = 0, *k*_1_ = 1, *k*_2_ = 50.

In order to have a better understanding of the interaction of the lamination angle and other influencing factors, 3D plots are presented to compare the buckling loads of the laminated plates with different aspect ratios, load ratios and temperature differences. Figure 18 shows the buckling load versus the lamination angle and aspect ratio, which shows that the aspect ratio is more sensitive to the buckling load than the lamination angle. In Figure 19, we find that the maximum buckling load reaches *θ* = 45°, which is independent of the load ratio. In addition, the lamination angle and compressive load coupling effects have a significant effect. With the temperature difference ranging from 0 °C to 80 °C, the strong dependence of the buckling load on the lamination angle is visible in Figure 20. In the considered temperature difference range, the buckling load increases with the lamination angle increase, then decreases.

## 5. Conclusions

The buckling load of functionally graded CFRP composite plates was investigated based on the classical laminate plate theory. The governing equations were derived based on the principle of virtual work and then solved by the Navier method. The results on the critical buckling load and the temperature effect of simply supported plates subjected to in-plane loading were obtained and discussed. The influence of the factors on the critical buckling of composite laminated plates was investigated in detail through parametric studies, such as the effect of the CF volume fraction, total number of layers, temperature, CF distribution pattern, geometrical dimension and lamination angle on the buckling properties of plates. Based on the results of those investigations, the following conclusions can be obtained:

(1) A larger $V_{F}^{*}$ corresponds to higher critical buckling loads; the buckling load decreases rapidly with the increase of the length-to-thickness ratio *k*_2_, then tends to become zero. The buckling loads are also significantly influenced by the lamination angle.
(2) X-shaped FG distribution is more effective than the other two distributions for reinforcing the plate for a higher buckling load, and compared to uniform distribution, the buckling load increased by 47%.
(3) A functionally graded composited plate with 10~15 individual layers stacked up can achieve a sufficient in-plane load.
(4) Critical buckling loads decrease with a temperature increase ranging from 0 °C to 80 °C.

## Figures and Tables

**Table 1:** Non-dimensional buckling loads *λ*_cr_ of rectangular laminates (0/90)_s_ under uniform compression and biaxial compression.

k	a/b	Theory	$E_{1}/E_{2}$				
			5	10	20	25	40
0	0.5	Reddy [43]	13.9000	18.1260	21.8780	22.8740	24.5900
		Present	13.9000	18.1265	21.8778	22.8738	24.5899
		Ansys	13.8666	17.6971	20.4871	20.9996	21.3372
	1.0	Reddy [43]	5.6500	6.3470	6.9610	7.1240	7.4040
		Present	5.6500	6.3470	6.9611	7.1238	7.4037
		Ansys	6.0568	6.6009	7.0363	7.1347	7.2598
	1.5	Reddy [43]	5.2330	5.2770	5.3100	5.3180	5.3320
		Present	5.2333	5.2768	5.3099	5.3182	5.3322
		Ansys	6.1796	5.8636	5.6220	5.5597	5.4452
1	0.5	Reddy [43]	11.1200	12.6940	13.9220	14.2480	14.7660
		Present	11.1200	12.6941	13.9222	14.2475	14.7661
		Ansys	11.6391	13.3583	13.7016	13.6693	13.3272
	1.0	Reddy [43]	2.8250	3.1740	3.4840	3.5620	3.7020
		Present	2.8250	3.1735	3.4806	3.5619	3.7019
		Ansys	3.0285	3.3005	3.5183	3.5675	3.6300
	1.5	Reddy [43]	1.6100	1.6240	1.6340	1.6360	1.6410
		Present	1.6103	1.6236	1.6338	1.6364	1.6407
		Ansys	1.7208	1.6935	1.6671	1.6590	1.6414

**Table 2:** Non-dimensional buckling loads for a simply supported square plate (a/h = 40).

Plate	Tsung-Lin Wu [40]	Present
Alumina	3.6498	3.6152
Aluminum	0.67	0.6660

**Table 3:** Material properties of the CFRP plate [46].

Material properties of fiber(carbon):			
$E_{11}^{F}$ = 230 GPa	$E_{22}^{F}$ = 23 GPa	$G_{12}^{F}$ = 9 GPa	$V_{F}^{*}=0.3$
$v_{12}^{F}$ = 0.2	$\alpha_{11}^{F}=-5.4\times 10^{-7}{℃}^{-1}$	$\alpha_{22}^{F}=1.008\times 10^{-5}{℃}^{-1}$	
Material properties of epoxy matrix:			
$E^{M}$ = (3.51 − 0.003 ΔT) GPa	$v^{M}=0.35$	$\alpha^{M}=45(1+0.001)\Delta T\times 10^{-6}{℃}^{-1}$	

**Table 4:** Effect of lamination scheme (*k*_0_ = 0, *k*_1_ = 1, *k*_2_ = 50).

Lay-Up	UD		FG_X	FG_O
	Ansys	Present		
[90/0/−45/45]_s_, [90/0/45/−45]_s_, [0/90/−45/45]_s_, [0/90/45/−45]_s_	50.78	51.46	69.11 ( + 34%)	34.61 (−33%)
[90/−45/45/0]_s_, [90/45/−45/0]_s_	59.41	61.25	73.48 (+20%)	41.43 (−32%)
[90/−45/0/45]_s_, [90/45/0/−45]_s_	54.63	57.99	77.40 (+33%)	37.62 (−35%)
[0/−45/90/45]_s_, [0/45/90/−45]_s_	54.63	57.99	79.14 (+36%)	37.62 (−35%)
[0/−45/45/90]_s_, [0/45/−45/90]_s_	59.41	61.25	81.87 (+34%)	41.43 (−32%)
[−45/90/45/0]_s_, [−45/0/45/90]_s_, [45/90/−45/0]_s_, [45/0/−45/90]_s_	63.26	71.04	103.94 (+46%)	38.72 (−45%)
[−45/45/90/0]_s_, [−45/45/0/90]_s_, [45/−45/90/0]_s_, [45/−45/0/90]_s_	74.24	77.57	113.97 (+47%)	41.73 (−46%)

Difference = 100%[*N*_cr_(FG) − *N*_cr_(UD)]/*N*_cr_(UD).

**Table 5:** Effect of average of CF volume fraction on the buckling loads (N/mm) for CFRP plates with different distribution patterns.

	$V_{F}^{*}$					
	0.05	0.1	0.15	0.2	0.25	0.3
Uniaxial compression						
Uniform	14.78	21.17(+43%)	27.59(+30%)	34.05(+23%)	40.55(+19%)	47.11(+16%)
FG-X	17.84	27.34(+53%)	36.93(+35%)	46.65(+26%)	56.51(+21%)	66.58(+18%)
FG-O	11.73	15.04(+28%)	18.37(+22%)	21.72(+18%)	25.09(+16%)	28.48(+14%)
Biaxial compression						
Uniform	7.39	10.58(+43%)	13.79(+30%)	17.02(+23%)	20.28(+19%)	23.56(+16%)
FG-X	8.92	13.67(+53%)	18.47(+35%)	23.32(+26%)	28.26(+21%)	33.29(+18%)
FG-O	5.86	7.52(+28%)	9.19(+22%)	10.86(+18%)	12.54(+15%)	14.24(+14%)

Slope = $\left|N_{cr}^{}\left(V_{F}^{*}\right)-N_{cr}^{}\left(V_{F}^{*}-0.05\right)\right|/N_{cr}^{}\left(V_{F}^{*}-0.05\right)$.

**Table 6:** Effect of aspect ratio and temperature difference for three distribution patterns.

Distribution Pattern	Temperature Difference	*k*_1_ = 1		*k*_1_ = 2.5	
		*γ_cr_*	Difference	*γ_cr_*	Difference
Uniform	Δ*T* = 0 ℃	5.35	−17.57%	5.39	−2.60%
	Δ*T* = 40 ℃	4.41		5.24	
FG-X	Δ*T* = 0 ℃	6.11	−12.11%	6.18	−1.78%
	Δ*T* = 40 ℃	5.37		6.07	
FG-O	Δ*T* = 0 ℃	3.95	−31.65%	3.93	−5.09%
	Δ*T* = 40 ℃	2.70		3.73	

## Data Availability

The datasets generated during and/or analyzed during the current study are available from the corresponding author on reasonable request.
